# Novel type of linear mitochondrial genomes with dual flip-flop inversion system in apicomplexan parasites, *Babesia microti* and *Babesia rodhaini*

**DOI:** 10.1186/1471-2164-13-622

**Published:** 2012-11-14

**Authors:** Kenji Hikosaka, Naotoshi Tsuji, Yoh-ichi Watanabe, Hiroe Kishine, Toshihiro Horii, Ikuo Igarashi, Kiyoshi Kita, Kazuyuki Tanabe

**Affiliations:** 1 Laboratory of Malariology, Research Institute for Microbial Diseases, Osaka University, 3-1 Yamadaoka, Suita, Osaka, 565-0871, Japan; 2 Department of Biomedical Chemistry, Graduate School of Medicine, The University of Tokyo, 7-3-1 Hongo, Bunkyo-ku, Tokyo, 113-0033, Japan; 3 Laboratory of Parasitic Diseases, National Institute of Animal Health, National Agriculture and Food Research Organization, Tsukuba, Ibaraki, Japan; 4 Department of Molecular Biology, Research Institute for Microbial Diseases, Osaka University, Suita, Osaka, Japan; 5 Department of Molecular Protozoology, Research Institute for Microbial Diseases, Osaka University, Suita, Osaka, Japan; 6 National Research Center for Protozoan Diseases, Obihiro University of Agriculture and Veterinary Medicine, Obihiro, Hokkaido, Japan

**Keywords:** Mitochondrial genome, *Babesia*/*Theileria*, Piroplasma, Apicomplexa, Flip-flop inversion

## Abstract

**Background:**

Mitochondrial (mt) genomes vary considerably in size, structure and gene content. The mt genomes of the phylum Apicomplexa, which includes important human pathogens such as the malaria parasite *Plasmodium*, also show marked diversity of structure. *Plasmodium* has a concatenated linear mt genome of the smallest size (6-kb); *Babesia* and *Theileria* have a linear monomeric mt genome (6.5-kb to 8.2-kb) with terminal inverted repeats; *Eimeria*, which is distantly related to *Plasmodium* and *Babesia*/*Theileria*, possesses a mt genome (6.2-kb) with a concatemeric form similar to that of *Plasmodium*; *Cryptosporidium*, the earliest branching lineage within the phylum Apicomplexa, has no mt genome. We are interested in the evolutionary origin of linear mt genomes of *Babesia*/*Theileria*, and have investigated mt genome structures in members of archaeopiroplasmid, a lineage branched off earlier from *Babesia*/*Theileria*.

**Results:**

The complete mt genomes of archaeopiroplasmid parasites, *Babesia microti* and *Babesia rodhaini*, were sequenced. The mt genomes of *B. microti* (11.1-kb) and *B. rodhaini* (6.9-kb) possess two pairs of unique inverted repeats, IR-A and IR-B. Flip-flop inversions between two IR-As and between two IR-Bs appear to generate four distinct genome structures that are present at an equi-molar ratio. An individual parasite contained multiple mt genome structures, with 20 copies and 2 – 3 copies per haploid nuclear genome in *B. microti* and *B. rodhaini*, respectively.

**Conclusion:**

We found a novel linear monomeric mt genome structure of *B. microti* and *B. rhodhaini* equipped with dual flip-flop inversion system, by which four distinct genome structures are readily generated. To our knowledge, this study is the first to report the presence of two pairs of distinct IR sequences within a monomeric linear mt genome. The present finding provides insight into further understanding of evolution of mt genome structure.

## Background

Mitochondria, organelles essential for energy transduction, are present in almost all eukaryotes and have their own genome. Like nuclear genomes, mitochondrial (mt) genomes vary considerably in size, structure, and gene content [1]. There are two major mt genome forms: circular and linear. Circular forms are present in animal mt genomes with sizes ranging from 15 kb to 20 kb and gene arrangements in the genomes are remarkably stable [2]. Some animal circular mt genomes are composed of more than two chromosomes or minicircles (e.g., the sucking louse *Pediculus humanus*, [3]). Circular forms are also found in higher-plant mt genomes. A higher-plant mt genome is characterized by a multipartite structure, which contains several subgenomic circular molecules with various organizational features, with sizes ranging from 200 kb to 2400 kb [4].

Linear mt genomes are found in diverse, unrelated organisms and have terminal inverted repeat (TIR) on both ends [5]. In some organisms, the mt genomes are divided into multiple chromosomes (e.g., the colorless green alga *Polytomella parva*, [6]), or several hundred chromosomes (e.g., the ichthyosporean *Amoebidium parasiticum*, [7]). In the phylum Apicomplexa, which includes important pathogens such as the causative agents of malaria (*Plasmodium*), coccidiosis (*Eimeria*), and piroplasmosis (*Babesia* and *Theileria*), the mt genome structure is also diverse. Monomeric linear mt genomes with TIR on both ends are found in the *Babesia* and *Theileria* genera [8]. The *Babesia*/*Theileria* mt genomes are from 6.6 kb to 8.2 kb in size and encode only three protein coding genes (cytochrome *c* oxidase subunit I *cox1* and III *cox3* and cytochrome *bcob*) in addition to 24 fragmented small subunit (SSU) and large subunit (LSU) ribosomal RNA (rRNA) sequences [8,9]. *Plasmodium*, closely related to *Babesia*/*Theileria*[10], has the minuscule 6-kb tandemly repeated linear or concatenated mt genome, which encodes the same three protein coding genes as *Babesia*/*Theileria*[11,12]. The gene arrangements and transcriptional direction are however different from *Babesia*/*Theileria*. Furthermore, SSU and LSU rRNA genes of *Plasmodium* are highly fragmented with 27 pieces [9,13] and the pattern of fragmentation differs from *Babesia*/*Theileria*[8,9]. *Eimeria*, which is distantly related to *Babesia*/*Theileria* and *Plasmodium*, possess a concatemeric form and contains the same three protein-coding genes and 20 rRNA gene fragments as *Plasmodium*[9,14,15]. A recent phylogenetic study suggests that a concatenated form appears to be the ancestral mt genome structure in the phylum Apicomplexa, with the monomeric linear form of *Babesia*/*Theileria* having evolved in the lineage [14]. We are interested in the evolution of linear mt genomes of *Babesia*/*Theileria*, and investigated mt genome structure of the rodent piroplasms, *Babesia microti* and *Babesia rodhaini*, which belong to archaeopiroplasmids group, a lineage which branched off earlier from *Babesia*/*Theileria*[16]. Results revealed that both *B. microti* and *B. rodhaini* have a monomeric linear mt genome, in which two pairs of unique IR sequences are present, and that flip-flop inversions in each pair of the IRs appear to generate four distinct mt genome structures.

## Results and discussion

### Mitochondrial genome organization

**Figure 1:**
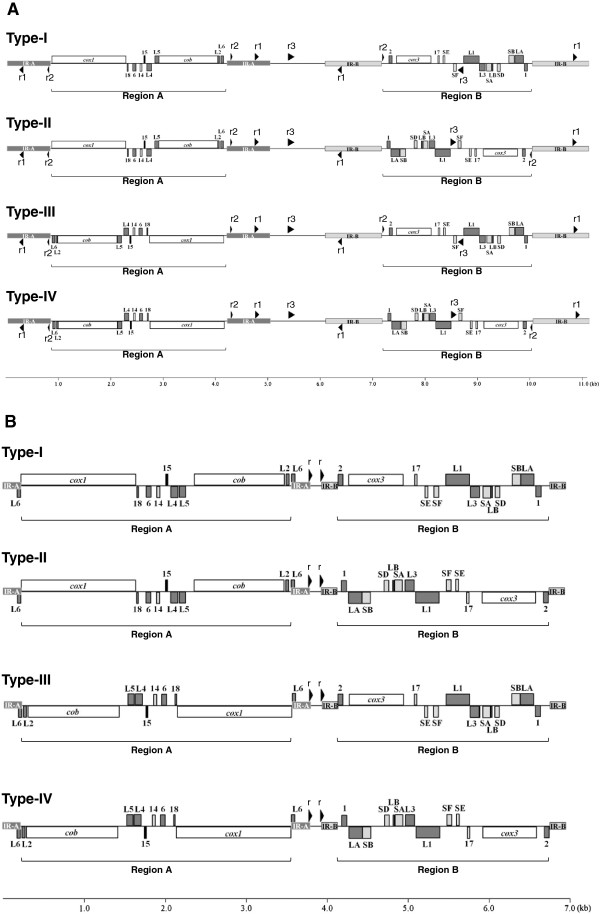
**Four distinct mitochondrial (mt) genome structures in *Babesia microti *(A) and *Babesia rodhaini *(B).** These mt genomes possess two pairs of inverted repeats, IR-A and IR-B. Genes shown above bold line are transcribed from left to right and those below from right to left. Light and dark gray blocks indicate fragments of small subunit (SSU) and large subunit (LSU) rRNA genes, respectively. Abbreviations: *cox1*, cytochrome *c* oxidase subunit 1 gene; *cox3*, cytochrome c oxidase subunit 3 gene; *cob*, cytochrome *b* gene*.* Black arrowheads, r1, r2 and r3 in the *B. microti* mt genome and r in the *B. rodhaini* mt genome, indicate short direct or inverted repeat sequences (see Additional file 2: Table S1 and Additional file 1: Figure S4).

**Figure 2:**
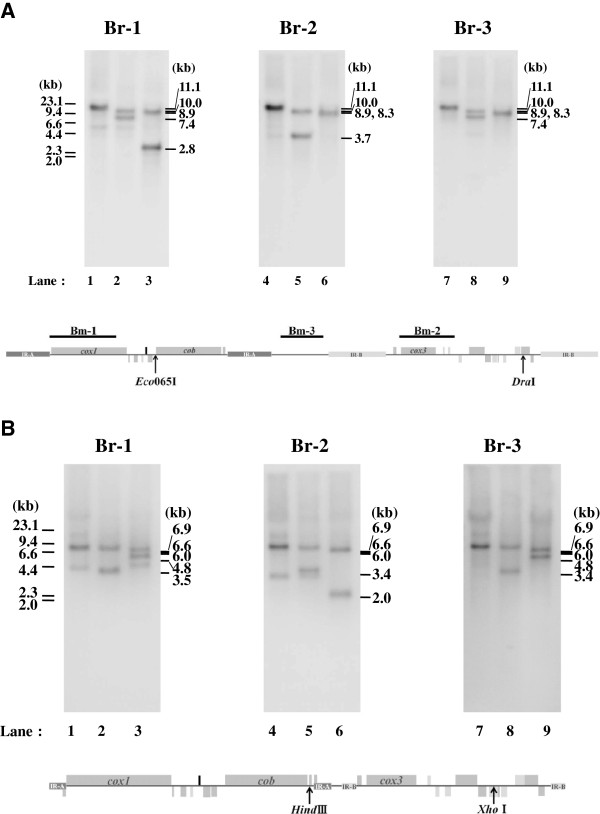
**Southern blot hybridization showing four distinct mitochondrial genome structures in *Babesia microti *(A) and *Babesia rodhaini *(B).** (**A**) Genomic regions of probes Bm-1, Bm-2 and Bm-3 and restricted enzyme sites of *Dra*I and *Eco*065I are shown in the lower map of the *B. microti* mt genome type-I (see Figure 1-A). Lanes 1, 4 and 7 for undigested DNA, lanes 2, 5 and 8 for *Dra*I-digested DNA, and lanes 3, 6 and 9 for *Eco*0651I-digested DNA. (**B**) Genomic regions of probes Br-1, Br-2 and Br-3 and restriction enzyme sites of *Hind*III and *Xho*I are shown in the lower map of the *B. rodhaini* mt genome type-I (see Figure 1-B). Lanes 1, 4 and 7 for undigested DNA, lanes 2, 5 and 8 for DNA digested with *Hind*III, and lanes 3, 6 and 9 for DNA digested with *Xho*I (lanes 3, 6 and 9).

We obtained the complete mt genome sequences of *B. microti* (Munich strain) and *B. rodhaini* (Australian strain). The *B. microti* mt genome was a monomeric linear molecule of 11.1 kb (Figures 1-A and 2-A) and contained three protein-coding genes, *cox1*, *cob* and *cox3*, and seven SSU and 12 LSU rRNA gene fragments (Figure 1-A). Unexpectedly, the *B. microti* mt genome possessed two pairs of long inverted repeats, inverted repeats A (IR-A) and B (IR-B). The nucleotide sequences of IR-A (817 bp) and IR-B (1082 bp) were dissimilar to each other. A 3.4-kb region between two IR-As contained *cox1*, RNA18, RNA6, RNA14, RNA15, LSU4, LSU5, *cob*, LSU2 and LSU6, and a 2.9-kb region between two IR-Bs contained RNA2, *cox3*, RNA17, SSUE, SSUF, LSU1, LSU3, SSUA, LSUB, SSUD, SSUB, LSUA and RNA1. The predicted secondary structures for fragments comprising the *B. microti* SSU and LSU rRNA, except for the LSU1-LSU6 whose secondary structures have been predicted in *Theileria parva* and *Babesia gibsoni*[8,17], are shown in Additional file 1: Figure S1-A. RNA15 is a transcript of unknown function in *Plasmodium falciparum* and *T. parva*[9]. An intervening region (1080 bp) between IR-A and IR-B does not appear to contain any gene or gene fragment. Searches for other repeat sequences identified additional three short repeats with lengths of 23, 65 and 103 bp in the *B. microti* mt genome (Figure 1-A and Additional file 2: Table S1).

Interestingly, the *B. microti* mt genome displayed four distinct genome structures, types I, II, III and IV (Figure 1-A). The four genome structures can be generated by two inversions: one is an inversion of a region containing the 3.4-kb region between the IR-As (here termed Region A) and the other an inversion of a region containing the 2.9-kb region between the IR-Bs (Region B). Southern blot hybridization with probe *cox1* (Bm-1) against undigested *B. microti* genomic DNA produced a clear signal at 11.1 kb (lane 1 in Figure 2-A). Hybridization against DNA digested with *Dra*I gave two bands at 10.0 kb and 7.4 kb (lane 2). Hybridization against *Eco*065I-digested DNA yielded two bands at 8.9 kb and 2.8 kb (lane 3). These results are consistent with the hypothesis that the four genome structures, types I, II, III and IV are generated by dual ‘flip-flop’ inversions of Region A and Region B. Thus, *Dra*I digestion produced 10-kb and 1.1-kb fragments (types I and III in Figure 1-A), and additionally produced 7.4-kb and 3.7-kb fragments (types II and IV, if Region B was inverted): and *Eco*065I digestion produced 8.9-kb, 2.2-kb (types I and II in Figure 1-A) and 8.3-kb and 2.8-kb fragments (types III and IV, if Region A was inverted). Hybridization with a *cox3* probe (Bm-2) revealed a clear signal at 11.1 kb against undigested genomic DNA (lane 4), two bands at 10.0 kb and 3.7 kb against DNA digested with *Dra*I (lane 5), and two bands at 8.9 kb and 8.3 kb against DNA with *Eco*065I (lane 6). Another *B. microti* probe from an intervening region (Bm-3) gave a band at 11.1 kb against undigested genomic DNA (lane 7), two bands at 10.0 and 7.4 kb against *Dra*I-treated DNA (lane 8), and two bands at 8.9 kb and 8.3 kb against *Eco*065I-digested DNA (lane 9). These results obtained with Bm-2 and Bm-3 are consistent with the idea of dual ‘flip-flop’ inversions.

The *B. rodhaini* mt genome (6.9 kb) also possessed two pairs of long inverted repeats, IR-A (220 bp) and IR-B (184 bp) and four distinct genome structures (types I, II, III and IV) (Figure 1-B). IR-A contained LSU6. A 3.3-kb region between two IR-As (Region A) contained *cox1*, RNA18, RNA6, RNA14, RNA15, LSU4, LSU5, *cob* and LSU2. A 2.6-kb region between two IR-Bs (Region B) contained RNA2, *cox3*, RNA17, SSUE, SSUF, LSU1, LSU3, SSUA, LSUB, SSUD, SSUB, LSUA and RNA1. The transcriptional direction of SSUE and LSU5 of the *B. rodhaini* mt genome is different from that of the *B. microti* mt genome. The predicted secondary structures for fragments comprising the *B. rodhaini* SSU and LSU rRNA, except for the LSU1-LSU6, are shown in Additional file 1: Figure S1-B. RNA15 seems to be functionally important since its nucleotide sequence is highly conserved among *B. microti*, *B. rodhaini*, *T. parva* and *P. falciparum* (Additional file 1: Figure S2). An intervening region (152 bp) between IR-A and IR-B does not appear to contain any gene and gene fragment. In addition to the two IRs, a pair of short direct repeat was identified (Figure 1-B and Additional file 2: Table S1).

As in the *B. microti* mt genome, the four genome structures of *B. rodhaini* can also be generated by dual flip-flop inversions of Region A and Region B. Thus, hybridization with Br-1 against undigested DNA produced a clear signal at 6.9 kb (lane 1 in Figure 2-B), as expected from the genome sequence. Hybridization against *Hind*III-digested DNA gave two bands at 6.6 and 3.5 kb (lane 2), the former being expected in types III and IV (Figure 1-B), and the latter expected in types I and II (Figure 1-B). Hybridization against *Xho*I-digested DNA yielded two bands at 6.0 and 4.8 kb, (lane 3), the former being expected in types I and III, and the latter expected in types II and IV. Hybridization with Br-2 yielded a band at 6.9 kb against undigested DNA (lane 4), two bands at 6.6 and 3.4 kb against *Hind*III-digested DNA (lane 5), and two bands at 6.0 and 2.0 kb against *Xho*I-digested DNA (lane 6). The intervening region probe (Br-3) gave a band at 6.9 kb against undigested DNA (lane 7), two bands at 6.6 and 3.4 kb against *Hind*III-digested DNA (lane 8), and two bands at 6.0 and 4.8 kb against *Xho*I-digested DNA (lane 9). These signals were consistent with the four genome structures (Figure 2-B).

**Figure 3:**
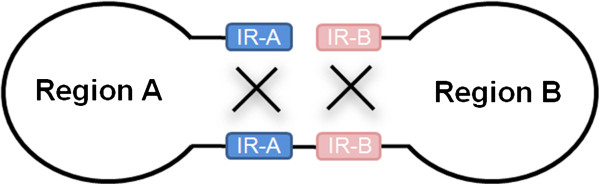
**A postulated mechanism for inversions of Region A and Region B.** Recombination between two IR-As (or two IR-Bs) produces an isomeric form characterized by a flip-flop of Region A (or Region B). Dual flip-flop inversions of IR-A and IR-B can generate four distinct genome structures.

All monomeric linear mt genomes characterized to date have a single pair of TIR on both ends [5]. Thus, to our knowledge this is the first study to show two pairs of distinct IR sequences. In both *B. microti* and *B. rodhaini*, dual flip-flop inversions of Region A and Region B appear to generate four mt genome structures. We postulate that the dual flip-flop inversions are mediated through recombination in palindromes of IR-A and IR-B (Figure 3). Recombination between a pair of IR-As (and IR-Bs) produces an isomeric form characterized by a flip-flop of Region A (and Region B), thus generating four distinct mt genome structures.

Flip-flop inversion of nuclear or organelle genomes have been found in some organisms. In the bacterium *Staphylococcus aureus*, large-scale inversion of its chromosome switches on or off different phenotypes, including the expression of dozens of genes [18]. The mt activity of the *Plasmodium* genus, which is closely related to the *Babesia* and *Theileria* genera, has been reported to be different between mosquito stages and vertebrate stages [19]. Therefore, it is likely that flip-flop inversions of the *B. microti* and *B. rodhaini* mt genomes may switch on or off expression of mt genes and gene fragments in their lifecycles.

### Transcription

RT-PCR using three separate primer sets targeting about 500-bp sequences of *cox1*, *cox3* and *cob* of *B. microti* gave the expected transcript size using cDNA but not RNA (Additional file 1: Figure S3). Similarly, expected PCR sized fragments were obtained using primers specific to *B. rodhaini* for *cox1*, *cox3* and *cob* (Additional file 1: Figure S3). Results confirm the transcription of the three protein-coding genes. We were unable to perform additional transcription analysis, such as a northern blotting, for 19 SSU and LSU gene fragments, due to extreme difficulties in obtaining an adequate amount of parasites from infected mice. Two reports of Kairo et al. [17] on the transcription of five LSU rRNA gene fragments (LSU1-LSU5) in *T. parva* and Hikosaka et al. [8] on the transcription of LSU6 in *B. gibsoni*, however, suggest that at least these six fragments are transcribed in *B. microti* and *B. rodhaini*.

### Estimations of the molar ratio of the four mt genome structures and mt genome copy number

We estimated the molar ratio of the four genome structures of *B. microti*. The intensity ratios of the two signals produced by hybridization of the Bm-1 probe with *Dra*I-digested DNA (lane 2 in Figure 2-A) and *Eco*065I-digested DNA (lane 3 in Figure 2-A) were 0.7 and 0.8 (Additional file 2: Table S2), respectively. Likewise, hybridization of the Bm-2 probe with *Dra*I-digested DNA (lane 5 in Figure 2-A), and the Bm-3 probe with *Dra*I-digested DNA (lane 8 in Figure 2-A) produced same signal intensities of 0.8 each. These suggest that a molar ratio of types-I, II, III and IV of the *B. microti* mt genome is approximately 1:1:1:1. We infer that the four distinct genome structures is generated from one parasite because the four genome structures was confirmed by Southern blot analysis for genomic DNA extracted from parasites derived from a single parasite cloned by limiting dilutions (data not shown). In addition, copy number analysis using Southern hybridization estimated about 20 copies of the mt genome per haploid nuclear genome. Taken together, these findings suggest that one parasite possesses four types of mt genome structure in *B. microti*.

In *B. rodhaini*, the molar ratio of the four mt genome structures (types I, II, III and IV) was also approximately 1:1:1:1. Thus, intensity ratio of the two signals produced in each case by hybridization of the Br-1 probe with DNA digested by *Hind*III or *Xho*I (lanes 2 and 3 in Figure 2-B), and of the Br-2 probe (lanes 5 and 6 in Figure 2-B), and of the Br-3 probe (lanes 8 and 9 in Figure 2-B) ranged from 0.9 to 1.2 (Additional file 2: Table S2). The equivalent molar ratio was confirmed by Southern blot analysis using DNA of *B. rodhaini* cloned by limiting dilutions (data not shown). Copy number of the *B. rodhiani* mt genome was estimated to be two to three copies per haploid nuclear genome. This suggests that one parasite has one to three types of mt genome structures, and four distinct mt genome structures may be generated during cell proliferation.

### Phylogeny

**Figure 4:**
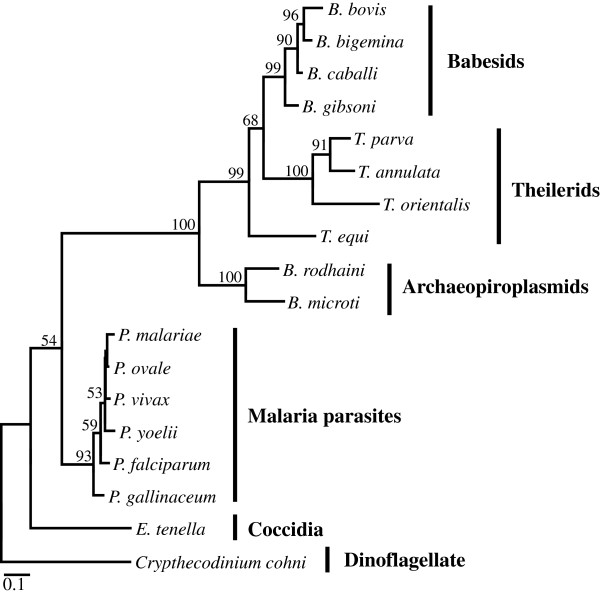
**The ML phylogenetic tree of mitochondrial protein coding genes, *cox1 *and *cob*, from 17 apicomplexans.***Crypthecodinium cohnii* was used as an outgroup. Concatenated amino acid sequences (696 sites) were used with 1,000 heuristic replicates under a Jones Taylor, and Thornton model [20] (α = 0.69) for constructing this tree. Numbers shown along nodes represent bootstrap values.

Both *B. microti* and *B. rodhaini* belong to an ancestral group of *Babesia*/*Theileria*, represented by the Archaeopiroplasmid group, according to phylogenetic analysis using 18S rRNA gene sequence [16]. The maximum likelihood (ML) tree inferred from concatenated COX1 and COB amino acid sequences (Figure 4) revealed a monophyletic relationship between Babesids, Theilerids and Archaeopiroplasmids with a bootstrap proportion (BP) value of 100%, clearly separated from a clade of *Plasmodium* species. *B. microti* and *B. rodhaini* were grouped into a clade (BP value 100%), which was located at the branch leading to the common ancestor of Babesids and Theilerids. These results indicate that the monomeric linear mt genomes found in the group of Babesids, Theilerids and Archaeopiroplasmids were generated specifically in this lineage.

## Conclusions

We found a novel linear monomeric mt genome structure in the rodent piroplasms, *Babesia microti* and *Babesia rodhaini*, equipped with dual flip-flop inversion system, by which four distinct genome structures are readily generated. Such a unique linear mt genome structure has not been known in not only apicomplexan parasites but also in other organisms. The present findings would provide insight into further understanding of evolution of mt genome structure.

## Methods

### Animals

Four-week-old female ICR mice were purchased from Japan SLC (Shizuoka, Japan) and housed in microisolator cages within a modified pathogen-free barrier facility at the Animal Resource Center for Infectious Diseases, Research Institute for Microbial Diseases, Osaka University. All animals had free access to food and water *ad libitum*, and all of the experimental procedures followed our institutional guidelines.

### DNA sequencing

*B. microti* (Munich strain) and *B. rodhaini* (Australian strain), were maintained by routine passage through mice. Infected blood was collected by cardiac puncture. Leukocytes were removed using Plasmodipur filters (EuroProxima, Arnhem, the Netherlands) [21]. Parasite genomic DNA was extracted using a QIAamp DNA Blood Mini Kit (Qiagen, Hilden, Germany) according to the manufacturer’s instructions. The mt genomes of *B. microti* and *B. rodhaini* were directly sequenced using specific primers (Additional file 2: Table S3-A). Primers were designed by aligning reported mt genome sequences of *Plasmodium falciparum* (DDBJ/EMBL/GenBank accession # M76611), *Plasmodium mexicanum* (EF079653), *Plasmodium yoelii* (MALPY00209), *Babesia bovis* (AB499088), *Theileria annulata* (NW_001091933), *Theileria equi* (AB499091) and *Theileria parva* (AB499089). Mt DNA was amplified in a 20 μl reaction mixture containing 0.2 μM each of forward and reverse primers, 400 μM each of dNTP, 1 unit of LA-Taq (Takara Bio, Shiga, Japan), 2 μl of 10 × PCR buffer, 2.5 mM of MgCl_2_, and 1 μl of genomic DNA. PCR conditions were as follows: initial denaturation at 94°C for 1 min, and amplification for 40 cycles of 94°C for 30 s, 55-68°C (depending on primers used) for 30 s, and 72°C for 1–6 min, depending on amplification size (1 min per kb), followed by a final extension at 72°C for 10 min.

Sequences of telomeric regions of the mt genomes of *B. microti* and *B. rodhaini* were determined by using the terminal deoxynucleotidyl transferase (TdT) tailing method [22] with minor modifications. Briefly, following initial denaturation of genomic DNA (150 ng) for 5 min at 95°C, the 3′-ends was tailed with cytosine for 30 min at 37°C in a reaction mixture containing 200 μM dCTP, 1 U of TdT (Takara Bio), 20 mM Tris–HCl (pH 8.4), 50 mM KCl, and 1.5 mM MgCl_2_, and then heat-inactivated at 65°C for 10 min. The first PCR was done in a 50-μl reaction mixture containing 2 μl of the tailed DNA fragments, 1.25 units of AmpliTaq DNA Polymerase (Applied Biosystems, Life Technologies, Carlsbad, CA), 2.5 mM MgCl_2_, 200 μM dNTPs, 0.4 μM of a mt genome-specific primer (Additional file 2: Table S3-B) and a selective anchor primer (5′-CTACTACTACTAGGCCACGCGTCGACTAGTACGGGGGGGGGGGGGGGG-3′). The PCR was performed by initial denaturation at 95°C for 2 min, and 40 cycles of 94°C for 30 s, 62°C for 3 min, followed by an extension step at 72°C for 10 min. For each sample, 1 μl of the first PCR products was used for the nested PCR amplification in a 50-μl reaction mixture as mentioned above, containing a nested primer (Additional file 2: Table S3-B) and a universal amplification primer (5′-CTACTACTACTAGGCCACGCGTCGACTAGTAC-3′). The second PCR was performed by initial denaturation at 95°C for 2 min, and 25 cycles of 94°C for 30 s, 62°C for 2 min, followed by an extension step at 72°C for 10 min. PCR products were purified using QIAquick PCR purification kit (Qiagen), and sequenced directly from two independent PCR products, using the BigDye® Terminator v3.1 Cycle Sequencing Kit (Applied Biosystems, Life Technologies) in an ABI 3130 Genetic Analyzer (Applied Biosystems, Life Technologies). Sequencing primers were designed to cover target regions in both directions. The sequences obtained in this study have been deposited in DDBJ/EMBL/GenBank with the following accession numbers, AB624353 – AB624356 (*B. microti* mt genome structures type-I to type-IV) and AB624357 – AB624360 (*B. rodhaini* mt genome structures type-I to type-IV).

### Gene annotation

Nucleotide sequences of the mt genomes from *B. microti* and *B. rodhaini* and their deduced amino acid sequences were aligned with reported sequences from *P. falciparum* (M76611), *B. bovis* (AB499088), *T. annulata* (NW_001091933), *T. equi* (AB499091) and *T. parva* (AB499089) by Clustal W [23] with manual correction. Protein-coding regions were inferred using previously annotated sequences from *T. parva* and *B. bovis*.

To identify putative rRNA genes, mt DNA sequences or annotated rRNA gene fragments from *B. bovis* (EU075182) and *T. parva* (Z23263) were used as a query under suggested algorithm parameters [24] in NCBI BLAST 2.2 [25]. In silico analysis was also performed with Probalign beta version 1.2 [26] and SSEARCH 3.5 [27] using known rRNA gene fragments and suggested advanced search options [24,26]. Newly identified candidate rRNA genes were, likewise, used as input sequences. Information from sequence alignments using CLUSTAL W [23] and putative base-pairings between fragments proposed for *T. parva* mt rRNA fragments [9,17] were used to determine the termini of candidate rRNA genes.

### Search for repeat sequences

Repeat sequences were searched using the REPFIND program (http://zlab.bu.edu/repfind/) [28] with cut-off of >20 nucleotides and a *P*-value < 0.0001. Inverted repeat sequences were searched using a ‘self against self’ BLASTN search [29] with cut-off of >20 nucleotides. Additional searches for repeats and inverted repeats were performed using GENETYX soft ware (Version 8; SDC, Tokyo, Japan).

### Southern blot hybridization

Genomic DNA of *B. microti*, either undigested or digested with *Dra*I or *Eco*065I, and that of *B. rodhaini*, either undigested or digested with *Hind*III or *Xho*I, were electrophoresed on 0.8% (w/v) agarose gels in TAE (40 mM Tris-acetate, 1 mM EDTA) and then transferred to a positively charged nylon membrane (Amersham Hybond-N+, GE Healthcare, Little Chalfont, England). Specifically amplified PCR products from *B. microti* and *B. rodhaini* genomic DNA (Additional file 2: Table S3-C) were labeled with digoxigenin-dUTP using the DIG High Prime DNA Labeling and Detection Starter Kit II (Roche Diagnostics, Rotkreuz, Switzerland). The DIG-labeled DNA probes were incubated with the nylon membrane, and blots were washed twice with 2 × SSC, 0.1% SDS and twice with 0.5 × SSC, 0.1% SDS, at 65°C for 15 min. Hybridization signals were detected using the Detection Starter Kit II. Chemiluminescence signals were quantitated using LAS-4000mini (GE Healthcare BioSciences AB, Uppsala, Sweden).

### RNA preparation and analysis

Transcription of *cox1*, *cox3* and *cob* in *B. microti* and *B. rodhaini* was analyzed by RT-PCR. Total RNA was extracted with RNeasy Mini Kit (Qiagen). Residual DNA in the RNA preparation was removed by DNase I treatment. cDNA synthesis and DNA amplification were carried out using specific primers (Additional file 2: Table S2-D) with PrimeScript® High Fidelity RT-PCR Kit (Takara Bio). RNA extracts that were not treated with reverse transcriptase gave no PCR products.

### Copy number estimation

Copy numbers of mt genomes of *B. microti* and *B. rodhaini* were estimated using dot blot hybridization. Briefly, DNA fragments of the mt genome and of the *B. microti* and *B. rodhaini* beta-tubulin genes (nuclear genome) were amplified by PCR using specific primers (Additional file 2: Table S3-C), and DNA amount was measured. Serial dilutions of control PCR products of known DNA amounts were dot-blotted onto a nylon membrane, following heat denaturation (99°C, 10 min). Genomic DNA were electrophoresed on agarose gels and then transferred to a nylon membrane. A PCR product specifically amplified from target regions of the mt genome and the beta-tubulin gene was labeled as described. Chemiluminescence signals were quantitated using LAS-4000mini.

### Phylogenetic analysis

Concatenated amino acid sequences of COX1 and COB (696 sites) from 17 apicomplexan parasites (Additional file 2: Table S4) were used for phylogenetic analysis. A free living dinoflagellate, *Crypthecodinium cohnii*[30,31], was included as an outgroup. COX3 were not used for phylogenetic analysis, due to very high divergence in *Babesia*/*Theileria* species [8]. We constructed the ML phylogenetic tree by the PROML program in PHYLIP version 3.68 [32]. CODEML program in PAML version 4.2 [33] was used to estimate the Γ shape parameter value α. Bootstrap analysis was done by applying PROML to 100 re-sampled datasets produced by SEQBOOT program in PHYLIP. BP values were calculated for internal branches of the ML-tree using CONSENSE in PHYLIP.

## Abbreviations

mt genome: Mitochondrial genome; kb: Kilobase; IR: Inverted repeats; TIR: Terminal inverted repeat; *cox1*: Cytochrome *c* oxidase subunit I gene; *cox3*: Cytochrome *c* oxidase subunit III gene; *cob*: Cytochrome *b* gene; LSU: Large subunit; rRNA: Ribosomal RNA; SSU: Small subunit; bp: Base pairs; ML: Maximum likelihood; BP: Bootstrap proportion; min: Minute; TdT: Terminal deoxynucleotidyl transferase; dCTP: Deoxycytidine 5′-triphosphate.

## Competing interests

The authors have declared that no competing interests exist.

## Authors’ contributions

KH carried out the molecular experiments. KT coordinated all experiments. KH, KK and KT contributed to the research design. KH and KT drafted the manuscript. NT and II provided parasite samples. YW analyzed data. HK and TH provided critical comments about this study. All authors read and approved the final manuscript.

## Supplementary Material

Additional file 1 This file contains Supplemental Figures S1-S4. Click here for file

Additional file 2 This file contains Supplemental Tables S1-S4. Click here for file
