# Supplementary material for: Novel type of linear mitochondrial genomes with dual flip-flop inversion system in apicomplexan parasites, Babesia microti and Babesia rodhaini
Source: BMC Genomics. 2012 Nov 14;13:622. doi: 10.1186/1471-2164-13-622 (PMC3546061; doi:10.1186/1471-2164-13-622)
Supplement: Additional file 1 — This file contains Supplemental Figures S1-S4. [file 1471-2164-13-622-S1.pdf]

Additional file 1: Supplementary tables

Table S1

Short direct and inverted repeat sequences in the mt genomes of *B. microti* and *B. rodhaini*

Species	Repeat	Similarity (%)	Nucleotide position*	Region
B. microti	65 bp repeat	65/65 (100%)	248 ← 311	IR-A
		65/65 (100%)	4679 → 4742	IR-A
		62/65 (96%)	6311 ← 6374	IR-B
		62/65 (96%)	10804 → 10867	IR-B
	23 bp repeat	23/23 (100%)	777 ← 799	IR-A
		23/23 (100%)	4191 → 4213	IR-A
		23/23 (100%)	7171 → 7193	Non-coding region
	103 bp repeat	103/103 (100%)	8607 → 8709	Non-coding region
		96/103 (94%)	5378 ← 5479	Intervening region
B. rodhaini	39 bp repeat	39/39 (100%)	3762 → 3800	IR-A + intervening region
		35/39 (90%)	3913 → 3951	IR-B + intervening region

* Nucleotides were positioned after the type-I structure of the mt genomes of *B. microti* and *B. rodhaini*

Table S2

Signal intensities of Southern blot analyses

Species	Probe	Lane	Signal intensity (a.u.)		Ratio*
			Upper signal	Lower signal	
B. microti	Bm-1	2	199142	234764	0.8
		3	268113	395847	0.7
	Bm-2	5	283589	363264	0.8
	Bm-3	8	176826	210086	0.8
B. rodhaini	Br-1	2	284106	308678	0.9
		3	245830	209233	1.2
	Br-2	5	230196	202346	1.1
		6	252830	246798	1.0
	Br-3	8	291410	257475	1.1
		9	242058	282028	0.9

$$\text{*Ratio} = \frac{\text{Upper signal intensity (a.u.)}}{\text{Lower signal intensity (a.u.)}}$$

Table S3
Primer sequences

Experiment	Species	Reaction	Probe	Gene	Name	Sequence
A. Genomic PCR	*B. microti*	-	-	-	BmicF17	5'-GTT TGC ATG ACA TTA TGA GCC TTC A-3'
					BmicR32	5'-CCT ATA GGA TTG TAG TCT TGT GCT A-3'
					BmicR3	5'-TTT GAA CCT CTT CAC CAC CCA AGT A-3'
					BmicR16	5'-GCA TTC TCT TGT AGA GTT GCA AGA A-3'
					BmicF27	5'-CTG GGA TCA TAA TCC CAT ACT AAG A-3'
					BmicR18	5'-AAG GCA ATA ATT ATC ACT ACC CCG A-3'
	B. rodhaini	-	-	-	BrodF11	5'-TTG CAT TAC CCA CAG GTA ATA AGA TT-3'
					BrodR11	5'-AGC AAC ATA GCT ACC ATA CCT GCA A-3'
					BrodR3	5'-CAA TAT CCG GCT ATA AAC GAG TTC A-3'
					BrodR4	5'-GCA CCA ATA CTT AAT ACA AAG TGG A-3'
					BrodR15	5'-TAC CCT GGG ATT CTA TAC CCA TAA T-3'
					BrodR10	5'-GTA TAT GTT CCG TTG TTT TAT GTT CC-3'
B. Sequencing of IR	*B. microti*	1st PCR	-	-	BmicR6	5'-CAC ATA CAC TTC TGG ATG TCC AAA G-3'
					BmicF21	5'-GAG CGT AGT AGA TAG CTA TTC ATC T-3'
			-	-	BmicR15	5'-CGA AAT GCC AGT ATG TAG CTA TGT A-3'
					BmicF30	5'-GCA GAC ATA GCT TGT TAC TGA ATC A-3'
		2nd PCR	-	-	BmicR9	5'-CCA GGC ATG ATG TTG AAG AAT ATC A-3'
					BmicR3	5'-TTT GAA CCT CTT CAC CAC CCA AGT A-3'
			-	-	BmicR16	5'-GCA TTC TCT TGT AGA GTT GCA AGA A-3'
					BmicF9	5'-GTG TAG ATA GCA GAT ATA TTC TAG TC-3'
	B. rodhaini	1st PCR	-	-	BrodR6	5'-GGA TCA CCA GAA TTT ACT GGA TCA-3'
					BrodF6	5'-CAA CAA GAT TTT CCC CCA CAT TGA-3'
			-	-	BrodF13	5'-ATT AGA GCT ATC CAT ACT GCC TCT A-3'
					BrodF2	5'-GCT TCC ATC GTC TTT CTC TTA GT-3'
		2nd PCR	-	-	BrodR7	5'-TCC TCC AAA CAA ACC GGG CAT GA-3'
					BrodF7	5'-ACT ACC ATT TTA CGG TAC ACT GAA-3'
			-	-	BrodF14	5'-CAG TTG CTG GAT AAG AAT CAA TAG G-3'
					BrodF12	5'-ACA TGG CAT AGA TAG CTT CCT ACG A-3'
C. Hybridization	*B. microti*	-	Bm-1		BmicF17	5'-GTT TGC ATG ACA TTA TGA GCC TTC A-3'
					BmicR4	5'-GAC ATC TGT TAG TGA TAA CTA CTT CA-3'
		-	Bm-2		BmicF27	5'-CTG GGA TCA TAA TCC CAT ACT AAG A-3'
					BmicR14	5'-ACG TCC TAC ATG AAC TTA GCA TCA A-3'
		-	Bm-3		BmicR13	5'-ACT GCT GCA AGC ACA AGT AGT AAC A-3'
					BmicF12	5'-GCA CAT CTG TTT ACG TTG CCA TTG A-3'
		-	BmTubulin		BmictubulinF2	5'-GAT AAC TAC GTT TTT GGT CAA TCT GGT-3'
					BmictubulinR2	5'-TGA ACA TTT CCT GTA TTG CAG TGG AGT-3'
	B. rodhaini	-	Br-1		BrodF9	5'-CCG AGG AGA ATT AGG AAG TAG TGG T-3'
					BrodR4	5'-GCA CCA ATA CTT AAT ACA AAG TGG A-3'
		-	Br-2		BrodR15	5'-TAC CCT GGG ATT CTA TAC CCA TAA T-3'
					BrodR22	5'-AGT CAA TAC GAA GTC GAA ACA AGG T-3'
		-	Br-3		BrodF21	5'-GAA CAA CGG TGG ATT AAA GCA TAG T-3'
					BrodR20	5'-TGT GCC TTC ACA AAA CCA CTT AAC T-3'
		-	BrTubulin		BrodtubulinF2	5'-GAT AAC TAT GTT TTT GGT CAA TCA GGA-3'
					BrodtubulinR2	5'-TGA ACA TTT CTT GAA TGG CAG TAG A-3'
D. RT-PCR	*B. microti*	-	-	*cox1*	BmicF38	5'-CTT TGG ACA TCC AGA AGT GTA TGT G-3'
					BmicR4	5'-GAC ATC TGT TAG TGA TAA CTA CTT CA-3'
				cox3	BmicF39	5'-TTC TTG CAA CTC TAC AAG AGA ATG C-3'
					BmicR15	5'-CGA AAT GCC AGT ATG TAG CTA TGT A-3'
				cob	BmicF21	5'-GAG CGT AGT AGA TAG CTA TTC ATC T-3'
					BmicR32	5'-CCT ATA GGA TTG TAG TCT TGT GCT A-3'
	B. rodhaini	-	-	*cox1*	BrodF10	5'-CGA TAA ACA ATT ACC TAC TTC ATG GT-3'
					BrodR5	5'-ACA CCC GTT GTA CCT CCT AAG GTA-3'
				cox3	BrodR17	5'-GTA AAC ACT TTG CAA GAA ATT AGC GA-3'
					BrodF13	5'-ATT AGA GCT ATC CAT ACT GCC TCT A-3'
				cob	BrodF19	5'-CTT CAT TTG CAA TAG GAT TTC TTG GT-3'
					BrodR11	5'-AGC AAC ATA GCT ACC ATA CCT GCA A-3'

Table S4

Parasite species used for phylogenetic analysis

Species	Stock/Strain	Host	Accession Number
Babesia microti	Munich strain	Rodent	AB624353 (this study)
Babesia rodhaini	Australian strain	Rodent	AB624357 (this study)
Babesia bovis	Miyama stock	Cattle	AB499088
Babesia bigemina	Kochinda stock	Cattle	AB499085
Babesia caballi	USDA strain	Horse	AB499086
Babesia gibsoni	NRCPD strain	Dog	AB499087
Theileria annulata	Ankara strain (C9)	Cattle	NW_001091933
Theileria parva	Muguga stock	Cattle	AB499089
Theileria orientalis	Ikeda stock	Cattle	AB499090
Theileria equi	USDA strain	Horse	AB499091
Plasmodium falciparum	C10 line	Human	M76611
Plasmodium vivax	Salvador I	Human	NC_007243
Plasmodium malariae	Uganda I	Human	AB354570
Plasmodium ovale	Nigeria II	Human	AB354571
Plasmodium yoelii	17XNL	Rodent	MALPY00209
Plasmodium gallinaceum	A8 strain	Bird	AB599930
Eimeria tenella	NIAH strain	Bird	AB564272
Crypthecodinium cohnii	WH-d	-	AF182643, AF403220
