# Supplementary material for: Novel type of linear mitochondrial genomes with dual flip-flop inversion system in apicomplexan parasites, Babesia microti and Babesia rodhaini
Source: BMC Genomics. 2012 Nov 14;13:622. doi: 10.1186/1471-2164-13-622 (PMC3546061; doi:10.1186/1471-2164-13-622)
Supplement: Additional file 2 — This file contains Supplemental Tables S1-S4. [file 1471-2164-13-622-S2.pdf]

Additional file 2: Supplementary figures S1-S4

Supplementary figure legends

Figure S1
SSU and LSU rRNA secondary structures of the mitochondrial genomes of *B. microti* (A) and *B. rodhaini* (B).

Figure S2
Nucleotide sequence alignments of *P. falciparum* RNA15 and its homologs from *B. microti*, *B. rodhaini* and *T. parva* [9]. Characters highlighted in gray indicate nucleotides differing from the *P. falciparum* sequence.

Figure S3
Transcription of the three protein coding genes, *cox1*, *cox3* and *cob*, of the mitochondrial genome of *Babesia microti* and *Babesia rodhaini*. For details, see the Methods section.

Figure S4
Nucleotide sequence of the 3′ region of IR-A, the intervening region and the 5′ region of IR-B of the *Babesia rodhaini* mt genome. Direct repeat sequence (39 bp) is boxed. Red and blue characters are sequence regions of IR-A and IR-B, respectively. Highlighted in grey is an intervening region. White characters in black background indicate nucleotides that are different between the two repeat sequences.

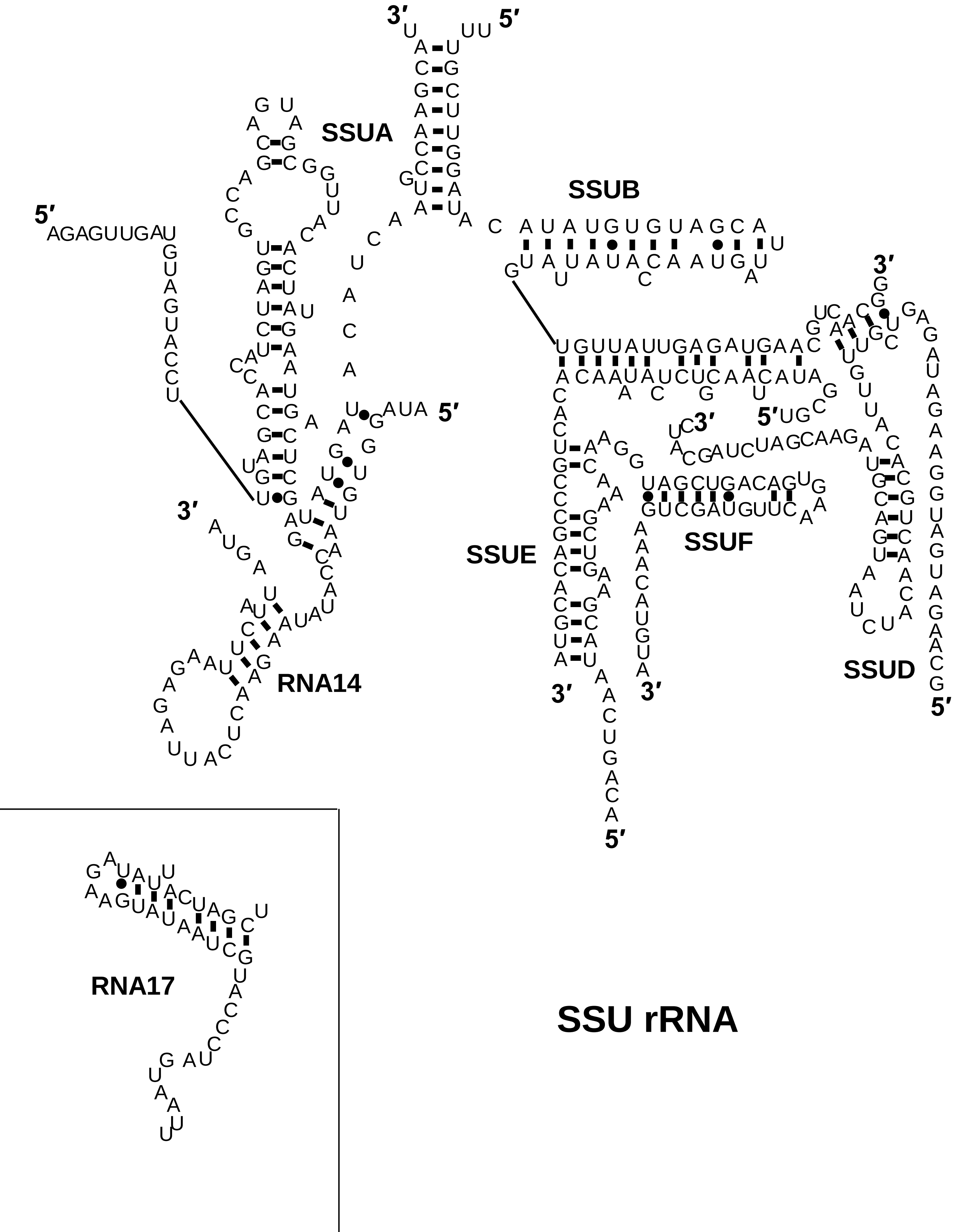

Figure S1-A

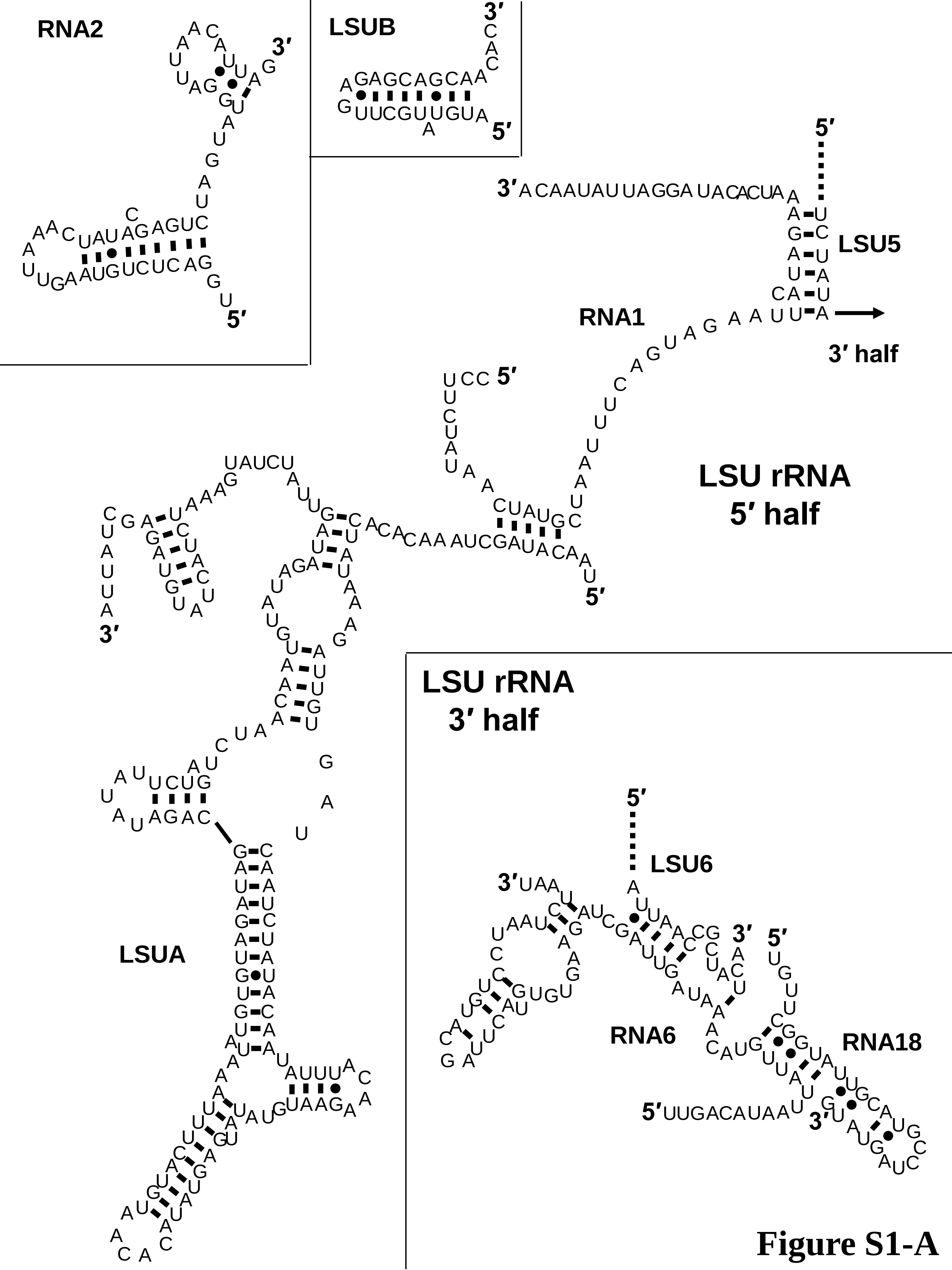

RNA2
LSUB
LSU5
RNA1
3′ half
LSU rRNA
5′ half
LSU rRNA
3′ half
LSU6
LSUA
RNA6
RNA18

Figure S1-A

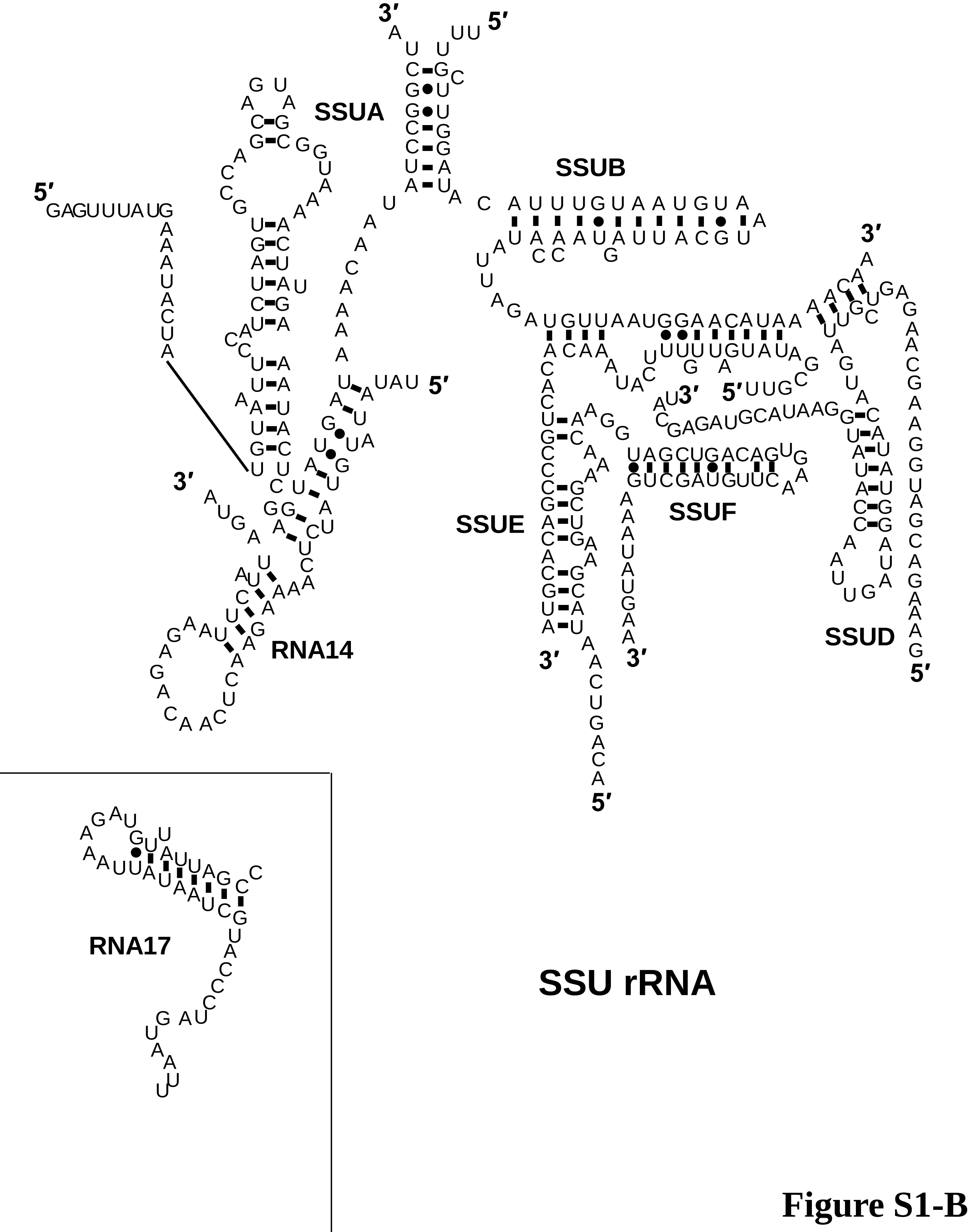

Figure S1-B

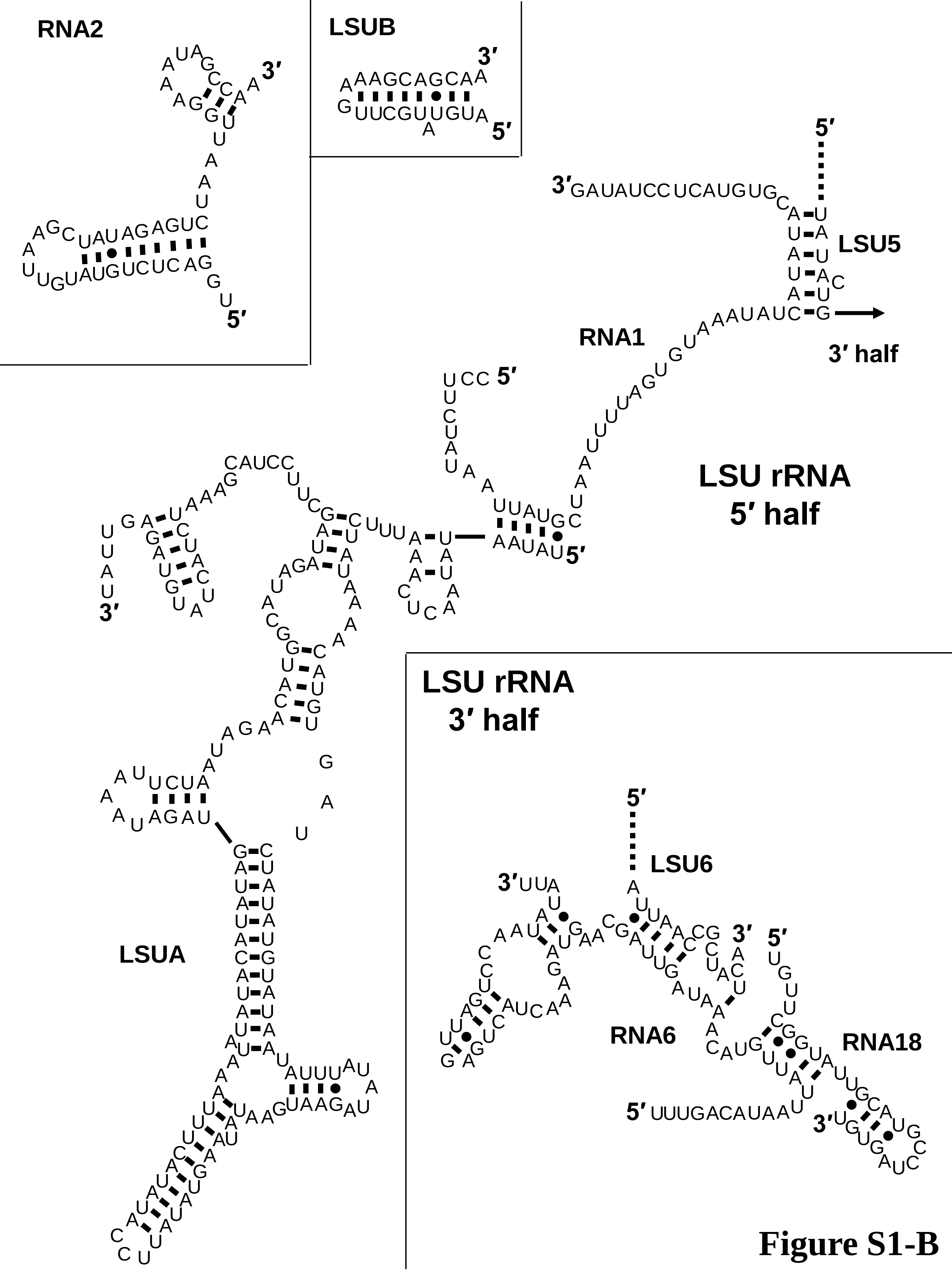
RNA2
LSUB
LSU5
RNA1
3′ half
LSU rRNA
5′ half
LSUA
LSU rRNA
3′ half
LSU6
RNA6
RNA18

Figure S1-B

```
P. falciparum   TG--GCGAGAAGGGAAGTGTGT-TTCCATAGAAA
B. microti      TACAGCGAGAAGGGAATTGTGC-TTCTATAGAAT
B. rodhaini     TA--GCGAGAAGGGAATTGTGC-TTCTATAGAAA
T. parva        TA--GCGAGATGGGAATTTTGCCTTCTATAGTTT
```

Figure S2

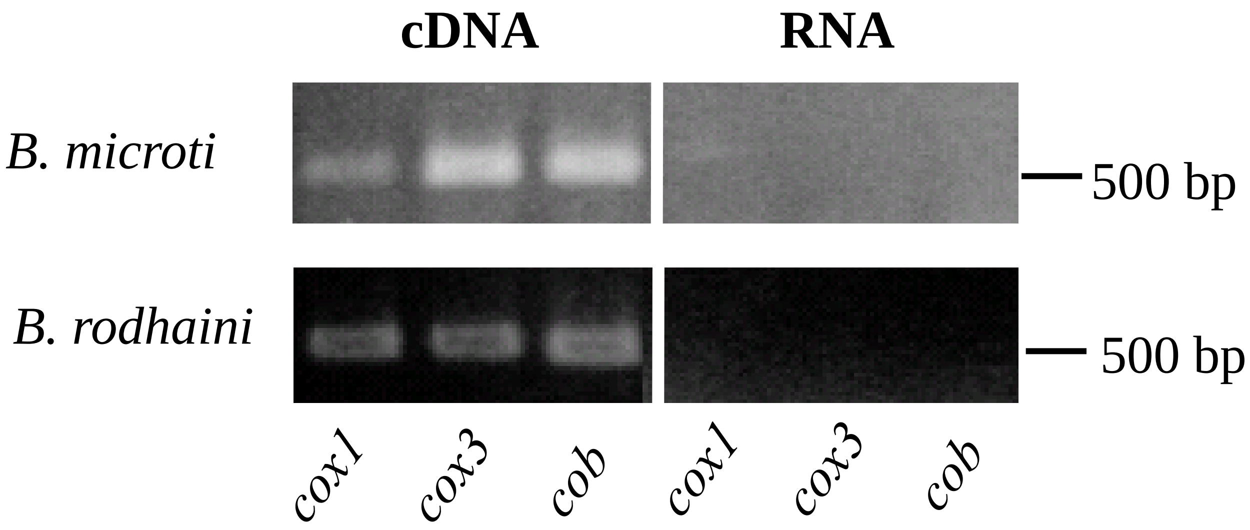
cDNA
RNA
B. microti
B. rodhaini
500 bp
500 bp
cox1
cox3
cob
cox1
cox3
cob

Figure S3

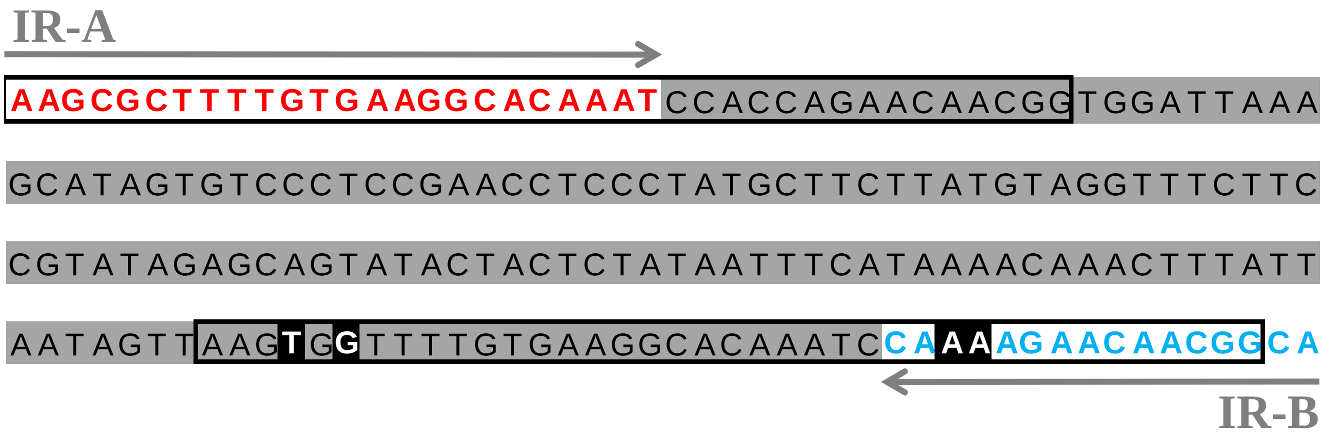
IR-A
AAGCGCTTTTGTGAAGGCACAAATCCACCAGAACAACGGTGGATTAAA
GCATAGTGTCCCTCCGAACCTCCCTATGCTTCTTATGTAGGTTTCTTC
CGTATAGAGCAGTATACTACTCTATAATTTCATAAAACAAACTTTATT
AATAGTTAAGTGGTTTTGTGAAGGCACAAATCCAAAAGAACAACGGCA
IR-B

Figure S4
